# Cell cycle arrest and induction of apoptosis in pancreatic cancer cells exposed to eicosapentaenoic acid in vitro.

**DOI:** 10.1038/bjc.1996.552

**Published:** 1996-11

**Authors:** P. B. Lai, J. A. Ross, K. C. Fearon, J. D. Anderson, D. C. Carter

**Affiliations:** Lister Research Laboratories, Department of Surgery, University of Edinburgh, Royal Infirmary, UK.

## Abstract

**Images:**


British Journal of Cancer (1996) 74, 1375-1383
© 1996 Stockton Press All rights reserved 0007-0920/96 $12.00

### Cell cycle arrest and induction of apoptosis in pancreatic cancer cells exposed to eicosapentaenoic acid *in vitro*

PBS Lai, JA Ross, KCH Fearon, JD Anderson and DC Carter

*Lister Research Laboratories, Department of Surgery, The University of Edinburgh, Royal Infirmary, Lauriston Place, Edinburgh EH3 9YW UK.*

**Summary** Eicosapentaenoic acid (EPA) has been shown to have an inhibitory effect on the growth of several pancreatic cancer cell lines *in vitro*. This study investigates the mechanism of growth inhibition and cytotoxicity of EPA on the pancreatic cancer cell line MIA PaCa-2. Cells were analysed for cell count, viability, cell cycle distribution and ultrastructural changes. There was a time- and dose-dependent decrease in cell count and viability in cultures of pancreatic cancer cells supplemented with EPA. Flow cytometric DNA anlaysis of MIA PaCa-2 cells incubated with EPA demonstrated the presence of sub $G_1$ populations corresponding to the presence of apoptotic cells and the blockade of cell cycle progression in S-phase and $G_2$/M-phase. The presence of apoptosis in EPA-supplemented cultures was further confirmed by DNA fragmentation and ultrastructural changes associated with apoptosis. Therefore, we conclude that EPA mediates its effect on the pancreatic cancer cell line MIA PaCa-2, at least in part, via cell cycle arrest and the induction of apoptosis.

**Keywords:** apoptosis; pancreatic cancer; eicosapentaenoic acid

Pancreatic cancer is currently the fifth most common cause of cancer death in the Western world. Despite recent improvements in diagnosis and staging, the prognosis remains poor with a median survival of 3-6 months (Carter, 1989). Unfortunately, surgical resection is rarely feasible, since most patients present with advanced inoperable disease. Although some groups have demonstrated slightly improved survival with conventional cytotoxic chemotherapy (Palmer *et al.*, 1994), there is, at present, no effective treatment for advanced pancreatic cancer. There is, therefore, a need to develop selective and relatively non-toxic treatment regimens directed at reducing the morbidity and mortality associated with pancreatic cancer.

In a number of experimental systems, certain polyunsaturated fatty acids (PUFAs) have been shown to have either a positive or negative influence (Holley *et al.*, 1974; Hudson *et al.*, 1993) on the growth of various tumour cell lines. The *n*-3 PUFA eicosapentaenoic acid (EPA) has potential as both an immunomodulator (Calder *et al.*, 1991; Tate *et al.*, 1988; DeMarco *et al.*, 1994; Soyland *et al.*, 1994) and as a direct anti-neoplastic agent (Hudson *et al.*, 1993; Beck *et al.*, 1991; Tisdale and Beck, 1991). We have previously examined the action of a variety of fatty acids on the growth of three human pancreatic cancer cell lines (MIA PaCa-2, PANC-1 and CFPAC). These studies demonstrated that, at concentrations of fatty acids ranging from 5 to 50 $\mu$M, EPA was one of the most effective inhibitors of cell growth (Falconer *et al.*, 1994). Studies with mice bearing the MAC-16 colon adenocarcinoma have demonstrated a marked inhibition of both tumour growth rate and associated cachexia following dietary supplementation with EPA (Tisdale and Beck, 1991; Tisdale and Dhesi, 1990). However, the exact mechanism of these observed effects is not clear. Mechanisms which may explain the inhibition of tumour growth by EPA have included increased lipid perodixation (Begin *et al.*, 1986; Lystad *et al.*, 1994) or eicosanoid-mediated cytotoxicity (Minoura *et al.*, 1988; Buckman *et al.*, 1991). It is also not known whether EPA exerts its effects by a reduction in cell proliferation or an increase in cell death.

Correspondence: JA Ross
Received 28 February 1996; revised 15 May 1996; accepted 17 May 1996

Cell death following exposure to EPA may be the result of necrosis, apoptosis or a combination of the two. Necrosis is the classical form of cell death characterised by membrane breakdown, loss of ion transport, cell swelling and lysis giving rise to an inflammatory response *in vivo*. Apoptosis (programmed cell death), on the other hand, is a programmed series of cellular events, whereby the cell ceases cell division and packages its internal components for removal without evoking an inflammatory response (Wylie *et al.*, 1980). The main features of apoptosis may vary depending on cell type but often include cell shrinkage, formation of apoptotic bodies, DNA fragmentation owing to activation of endogenous endonucleases (Arends and Wylie, 1991; Cohen and Duke, 1984; Cohen, 1991) and the appearance of sub $G_1$ or hypodiploid peaks on flow cytometric DNA analysis (Bryson *et al.*, 1994; Telford *et al.*, 1994).

This study examines the mechanisms of growth inhibition by EPA on the pancreatic cancer cell line MIA PaCA-2. We describe the induction of cell cycle arrest and apoptosis in MIA PaCa-2 upon exposure to EPA *in vitro*.

### Materials and methods

### *Reagents*

Palmitic acid (PA), eicosapentaenoic acid (EPA), fatty acid-free bovine serum albumin (BSA) and propidium iodide were purchased from Sigma (Sigma, Poole, Dorset, UK). Fatty acids were complexed to BSA according to the method described previously (Falconer *et al.*, 1994) and 1 mM stock solutions were stored at $-20°C$ until use.

### *Cell line*

The human pancreatic cancer line, MIA PaCa-2, was obtained from the European Collection of Cell Cultures (PHLS Centre for Applied Microbiology and Research, Porton Down, UK). Cells in exponential growth were used for experiments and were grown in Dulbecco's modified Eagle medium (DMEM) supplemented with 5% fetal calf serum (FCS) (MB Meldrum, UK), penicillin 50 units $ml^{-1}$ and streptomycin 50 $\mu g$ $ml^{-1}$ (Life Technologies, Inchinan, UK) and 1 mM glutamine (Life Technologies) in a 95% air/5% carbon dioxide humidified incubator.

*Cell count and viability*

About $10^6$ cells were incubated in 25 $cm^2$ cell culture flasks with 10 ml of standard medium and were allowed to adhere for 24 h before supplementation with either BSA, PA or EPA (at concentrations of 10 $\mu$M, 30 $\mu$M and 50 M). PA, a saturated fatty acid which has been shown (Falconer *et al.*, 1994) to have no effect on the growth of pancreatic cancer cell lines, was chosen as a control fatty acid. BSA was also used as another control as both PA and EPA used in the experiments were albumin complexed. Parallel cultures were harvested at 24, 48 and 72 h after supplementation with BSA or fatty acids. All cells (adherent and non-adherent) were collected and resuspended in phosphate-buffered saline (PBS) (pH 7.2) before determining the cell number and viability with propidium iodide exclusion on a Coulter XL flow cytometer (Coulter Electronics, Luton, UK). Briefly, to 1 ml of cell suspension 100 $\mu$l of 0.025% propidium iodide solution was added 10 min before analysis and samples were analysed where non-viable cells were read as those cells positive for propidium iodide. The cell count and viability were also assessed using trypan blue exclusion under a microscope.

*Electron microscopy*

Cells were trypsinised from flasks, washed by centrifugation in PBS and the cell pellets fixed in 2.5% glutaraldehyde in 0.1 M phosphate buffer. Cell pellets were processed as described previously (Middleton *et al.*, 1992).

*Cell cycle experiments*

Parallel cultures were prepared in the same way as cell growth experiments so that samples were harvested at 12, 24, 36, 48, 60 and 72 h after supplementation with PA (30 $\mu$M and 50 $\mu$M) or EPA (30 $\mu$M and 50 $\mu$M). Cell cycle distribution and apoptosis were determined as described by Telford *et al.* (1994). In brief, 500 $\mu$l of nuclear staining solution (containing 50 mg of propidium iodide, 3 ml of Nonidet P40 and 1 mg of sodium citrate per litre) and 500 $\mu$l of RNAase (500 units $ml^{-1}$) were added to $10^6$ cells. The cells were analysed after incubation in the dark at 20°C for 30 min. Total DNA content per cell was determined by analysis of fluoresence at 488 nm by using a Coulter XL flow cytometer (Coulter Electronics). A minimum of $10^4$ cells per sample were analysed. Data were displayed as the total number of cells in each of the 1024 channels of increasing propidium iodide fluorescence intensity, and the resulting histograms were analysed with WinMDI analysis, Version 2 (J Cotter@Scripps, USA, Windows 3.1 multiple document interface flow cytometry application) and Coulter DNA analysis software.

*Analysis of DNA fragmentation*

DNA fragmentation was analysed by agarose gel electrophoresis. Cells ($5 \times 10^6$) were harvested, washed, centrifuged at 4000 ***g*** and the supernatant removed. MIA PaCa-2 cells exposed to 50 $\mu$M of EPA for 24, 48 or 72 h, untreated MIA PaCa-2 cells and Jurkat cells induced to undergo apoptosis by incubation in the presence of cyclohexamide were prepared. Following centrifugation, cell pellets were incubated in 500 $\mu$l of lysis buffer [50 mM Tris-HCl, pH 8.0, containing 1 mM EDTA, 0.25% NP40 and 0.1% RNAase A (Sigma)] for 30 min at 37°C. A sample of 50 $\mu$l of 10 mg $ml^{-1}$ proteinase K (Boehringer-Mannheim, Lewes, UK) was then added and the incubation continued for a further 30 min at 37°C. After incubation, 20 $\mu$l of the product and 3.6 $\mu$l of loading buffer (blue-orange; Promega, Southampton, UK) were mixed and added to each well of a 2% agarose gel containing 5 $\mu$g $ml^{-1}$ ethidium bromide. Electrophoresis was carried out at 10 V $cm^{-1}$. Background fluorescence caused by unbound ethidium bromide was reduced by soaking the stained gel in 1 mM magnesium

**Figure 1** (**a**) Cell count ($\times 10^6$) of MIA PaCa-2 cells incubated with 10 $\mu$M (○), 30 $\mu$M (□) or 50 $\mu$M (△) EPA compared with cells incubated with 50 $\mu$M PA (■). Counting was performed using a Coulter flow cytometer set to aspirate a constant volume. Bars are mean ± s.e.m. Statistical comparisons were made between EPA and the appropriate PA control using Student's *t*-test ($*P<0.001$). (**b**) Viability of MIA PaCa-2 cells incubated with 10 $\mu$M (○), 30 $\mu$M (□) or 50 $\mu$M (△) EPA compared with cells incubated with 50 $\mu$M PA (■) as determined by propidium iodide exclusion. Bars are mean ± s.e.m. Statistical comparisons were made between EPA and the appropriate PA control using Student's *t*-test ($*P<0.05$).

sulphate for 20 min at room temperature before inspection using an ultraviolet transilluminator (Jencons Scientific, UK). The gel was photographed using Polaroid type 665 film.

*Statistics*

Statistical analysis was carried out using a two-tailed Student's *t*-test, and significant differences were assumed

**Figure 2** Two-parameter flow cytometry profiles of size (forward scatter) (FS) *vs* propidium iodide (PI) (red fluorescence). Cells were grown in medium supplemented with 50 μM PA for 24 h. (**b**), 48 h (**d**) and 72 h (**f**), or 50 μM EPA for 24 h (**a**), 48 h (**c**) and 72 h (**e**). The percentage of PI-dim apoptotic cells increased with time of exposure to 50 μM EPA (4% at 48 h and 10% at 72 h) as did the population of dead cells (23% at 48 h and 26% at 72).

when the chance of differences arising from a sampling error was less than 1 in 20 (i.e. $P<0.05$). The analysis of DNA histograms used the DNA histogram model of Bagwell *et al.* (1979) in which the S-phase is modelled with Gaussian curves. The fitting of the data to the model is accomplished with a standard least-squares multiple regression routine.

**Figure 3** Cell monolayers incubaed in the presence of PA at 50 μM (**a**) or EPA at 50 μM (**b**) for a period of 72 h. Using phase-contrast microscopy, the cells exposed to PA have maintained a monolayer, while those grown in EPA are rounded up and shrunken. In addition, in cells grown in the presence of EPA, there are variable amounts of vesicles in the cytoplasm (×230).

### Results

### *Effect of EPA on cell number and viability*

In parallel cultures with cells growing in standard medium alone or supplemented with either BSA (10 μM, 30 μM and 50 μM) or PA (10 μM, 30 μM and 50 μM), there was no significant difference in the cell count and viability at all time points (data not shown). For clarity only the data for 50 μM PA is plotted in the graphs.

Supplementation of culture medium for 72 with EPA at concentrations of 10 μM, 30 μM and 50 μM reduced cell growth (Figure 1a) ($P<0.001$, $P<0.001$ and $P<0.001$ respectively), when compared with cells in medium supplemented with PA. This effect occurred in a time- and dose-dependent manner (Figure 1a). Cells grown in medium supplemented with PA (Figure 1b) showed no significant decrease in viability throughout the experiment as determined by propidium iodide exclusion. Cells grown in medium supplemented with 10 μM EPA (Figure 1b) showed a slight decrease in viability but this only reached statistical significance after 48 h of exposure (97.97% *vs* 94.40% after 72 h, $P=0.01$). A more marked reduction in the viability of cells exposed to EPA at 30 μM and 50 μM (Figure 1b) was noted after only 24 h of exposure. The viability of such cells was reduced to 80.33% ($P<0.001$) and 74.53% ($P<0.001$) after 72 h of exposure to EPA at 30 μM and 50 μM respectively.

### *Flow cytometry*

At zero time all cells excluded propidium iodide and exhibited similar forward scatter (data not shown). In cells harvested after 24 h of exposure to PA or EPA, at concentrations of 10 μM or 30 μM, there was no difference in two-parameter plots of size (forward scatter) against propidium iodide (red fluorescence) (data not shown). However, when cells grown in the presence of 50 μM PA or 50 μM EPA for 24 h were compared, there was a small population (10%) of dead cells (propidium bright) in the cells from EPA cultures (Figure 2a) and no such population in cells from PA cultures (Figure 2b). In cells exposed to 50 μM EPA for 48 h, there were three distinct populations of cells observed in the two-parameter plots of propidium iodide (PI) *vs* forward scatter (FS). These three populations represented in the live cells (PI-negative), the dead cells (PI-bright) and the apoptotic cells (PI-dim) (Figure 2c). The PI-dim and PI-bright cells also exhibited reduced forward scatter. The PI-dim and PI-bright cells were absent from two parameter plots of cells grown in the presence of 50 μM palmitic acid (Figure 2d). The percentage of PI-dim apoptotic cells (Figure 2e) increased with time of exposure to 50 μM EPA (4% at 48 h and 10% at 72 h) as did the population of dead cells (23% at 48 h and 26% at 72 h). The distinct apoptotic population and the population of dead cells were not observed in cells exposed to 50 μM palmitic acid over a period of 72 (Figure 2f).

### *Effect of EPA on cell morphology and ultrastructure*

On inspection of cell cultures using phase-contrast microscopy, the cells grown in medium without supplement or in medium supplemented with BSA or PA (Figure 3a) had formed an adherent monolayer, while a proportion of those grown in EPA (Figure 3b) were round and shrunken, a feature suggestive of detachment from the surface of the culture flask. These features were most obvious in cells grown in medium supplemented with 50 μM EPA (Figure 3b), but were also observed in cultures containing 30 μM and 10 μM EPA (data not shown). In addition there were variable amounts of vesicles in the cytoplasm of cells grown in the presence of EPA as well as vesicles in close proximity to both the cell membrane and the nuclear membrane (Figure 3b).

Ultrastructural changes associated with apoptosis were observed (Figure 4a, b and c) with the earliest indicators of apoptosis being slight nuclear margination and small coarse aggregates of chromatin throughout the nucleus.

**Figure 4** Ultrastructural appearance of MIA PaCa-2 cells in the presence of 50 $\mu$M eicosapentaenoic acid (**a**). The earliest indicators of apoptosis are slight nuclear margination and small coarse aggregates of chromatin throughout the nucleus. There are pronounced changes in nucleolar organisation ($\times$ 7032). (**b**) Some cells exhibiting the changes noted above contain large globules with their cytoplasm ($\times$ 8438). (**c**) Portion of cell containing a phagocytosed apoptotic body (arrow). Membrane structures and condensed nuclear material can still be discerned within the apoptotic body ($\times$ 7032). (**d**) Clumping of chromatin in a later stage apoptotic cell ($\times$ 14420). (**e**) Intranucleosomal DNA fragmentation is evident in DNA isolated from MIA PaCa-2 cells exposed to 50 $\mu$M EPA for 24 h (lane 1), 48 h (lane 2) or 72 (lane 3). Intranucleosomal DNA fragmentation was absent from DNA from untreated cells (lane 4).

**Figure 5** DNA histograms of MIA PaCa-2 cells grown in the presence of (**a**) 50 μM PA or (**b**) 50 μM EPA for 72 h. DNA histograms demonstrate the absence of a hypodiploid peak in preparations from cells grown in PA-supplemented medium (**a**) and the presence of a hypodiploid peak in preparations from cells grown in EPA-supplemented medium (**b**).

There were pronounced changes in nucleolar organisation with the appearance of condensed aggregates of chromatin around the periphery and within the nucleolus (Figure 4a). The cytoplasm and cytoplasmic organelles showed no changes in morphological appearance, although large globules were occasionally seen within the cytoplasm of cells grown in the presence of EPA (Figure 4b). Flow cytometry confirmed the presence of lipid accumulation, by Nile red staining (Greenspan *et al.*, 1985), in the cells exposed to EPA when compared with those grown in the absence of EPA (data not shown). The nulcear membrane remained intact. Portions of apoptotic cells inside phagocytic vacuoles were occasionally observed within the cytoplasm of neighbouring MIA PaCa-2 cells (Figure 4c). Membraneous structures and condensed nuclei or nuclear material could still be discerned within the apoptotic bodies. The nucleus of cells in the later stages of apoptosis were occasionally observed and exhibited condensation of chromatin around the nuclear periphery and within the coarse nuclear matrix (Figure 4d).

*DNA fragmentation*

In order to determine the ability of EPA to induce apoptosis, soluble DNA was extracted from cells exposed to 50 μM EPA for 24, 48 or 72 h, from cells grown in the absence of EPA or the presence of palmitic acid and from Jurkat cells induced to undergo apoptosis in the presence of cyclohexamide (data not shown). A clear pattern of intranucleosomal DNA fragmentation was evident (Figure 4e) in DNA isolated from MIA PaCa-2 cells exposed to 50 μM EPA for 24, 48 or 72 h. Intranucleosomal DNA fragmentation was absent from DNA from untreated cells (Figure 4e) or from cells grown in 50 μM palmitic acid (data not shown).

*Effect of EPA on cell cycle*

DNA histograms prepared from cells at 0, 12, 24, 36, 48, 60 (data not shown) and 72 h (Figure 5) demonstrated the absence of a hypodiploid peak in preparations from cells grown in PA-supplemented medium. Hypodiploid peaks were present in preparations from cells grown in medium supplemented with 50 μM EPA after 36 h. The presence of a hypodiploid peak was most obvious in cells grown in 50 μM EPA (Figure 5) for 72 h but was also seen in cells grown in 30 μM EPA (data not shown). The percentages of the sub $G_1$ peaks after 48, 60 and 72 h in culture supplemented with 50 μM EPA were 4%, 6% and 11% respectively.

The effect of PA and EPA on the distribution of cells in the cell cycle at different concentrations and duration of exposure is shown in Figure 6. In cells exposed to PA at 30 μM or 50 μM, there was a progressive increase in the proportion of cells in $G_0/G_1$ phase (Figure 6a) and a progressive decrease in the proportion of cells in S-phase (Figure 6b), while the proportion of cells in $G_2$/M-phase remained static throughout the time of culture (Figure 6c). There was no obvious hypodiploid or sub $G_1$ peak present.

For cells exposed to EPA at 30 μM, a different pattern was observed in that the proportion of cells in $G_0/G_1$ did not increase to the same degree as the PA-supplemented group (Figure 6a) and the proportion of cells in S-phase did not decrease (Figure 6b) to the same degree as the PA-supplemented group. The proportion of cells in $G_0$/M-phase followed a similar pattern to those supplemented with PA (Figure 6c). At an EPA concentration of 50 μM, there was an even further reduction in the proportion of cells in $G_0/G_1$ phase (Figure 6a), while the proportion of cells in S-phase remained unchanged (Figure 6b). The proportion of cells in $G_2$/M-phase again remained static (Figure 6c).

### Discussion

EPA has previously been shown to inhibit the growth of pancreactic cancer cell lines (Falconer *et al.*, 1994). In the current study, we have observed similar inhibitory effects with EPA supplementation. When MIA PaCa-2 cells were exposed to EPA at 30 μM and 50 μM, there was a significant reduction in the total cell number as well as in the viability of cells (Figure 1a and b). The growth inhibition and cytotoxic effect were both time- and concentration-dependent. Furthermore, by using the propidium iodide exclusion method, we can identify a distinct population of apoptotic cells (Figure 2c and e) in cultures supplemented with EPA, which are distinct from the

**Figure 6** The effect of supplementation of medium with 30 μM and 50 μM PA or EPA on the distribution of cells in the cell cycle following different times of exposure. In cells exposed to PA at 30 μM (□) or 50 μM (◇), there was a progressive increase in the proportion of cells in $G_0/G_1$ phase (**a**) and a progressive decrease in the proportion of cells in S-phase (**b**) on serial DNA histograms, while the proportion of cells in $G_2$/M-phase remained relatively static (**c**). For cells exposed to EPA at 30 μM (○), a different pattern was observed in that the proportion of cells in $G_0/G_1$ did not increase (**a**) to the same degree as the PA-supplemented group and the proportion of cells in S-phase did not decrease (**b**) to the same degree as the PA-supplemented group. At an EPA concentration of 50 μM (△) the proportion of cells in $G_0/G_1$ phase decreased with time (**a**) while the proportion of cells in S-phase (**b**) and $G_2$/M-phase (**c**) remained static.

population of live cells and from the population of dead cells. The propidium-dim cells may represent the early stages of apoptosis where the cell membrane can still exclude propidium but reduced repair mechanisms allow trapping of propidium in pits on the cell surface membrane. The presence of dead cells in the two-parameter plots (Figure 2a, c and e) represent those cells in the very late stages of apoptosis with reduced size, which can no longer exclude propidium iodide (Arends and Wylie, 1991; Telford *et al.*, 1994; Bryson *et al.*, 1994).

The presence of apoptotic cells can also be demonstrated in DNA histograms by the appearance of a sub $G_1$ peak, which is an established indicator of apoptosis (Bryson *et al.*, 1994; Telford *et al.*, 1994). In cells exposed to 50 μM EPA for 72 h, there was a sub $G_1$ peak, which compromised approximately 11% of the population (Figure 5). Apoptosis was further confirmed in cells exposed to EPA by the distinctive morphological and ultrastructural changes of apoptosis (Figure 4a to d), and by the presence of laddering on ethidium-stained DNA gels (Figure 4e) (Cohen, 1991). This is, therefore, the first demonstration that EPA can induce apoptosis in pancreatic tumour cells.

In cells exposed to 50 μM EPA, there were progressively fewer cells in $G_0/G_1$ phase and more cells in S-phase, while the proportion of cells in the $G_2$/M-phase remained static (Figure 6). This may represent an increase in cellular proliferation or an arrest of the cell cycle in S-phase, which prevented cells from progressing towards mitosis. An increase in cellular proliferation would appear improbable as both the total number of cells and the viability were decreased rather than increased. The data is more consistent with cell cycle arrest and the accumulation of cells in S-phase. This is also consistent with previous findings of reduced thymidine uptake in cells exposed to EPA (Falconer *et al.*, 1994). Unfortunately, it is not possible to determine whether the cells were blocked in S-phase or early $G_2$/M-phase from this series of experiments because of the recognised difficulties in distinguishing cells in S-phase and early $G_2$/M-phase on DNA histograms (Ormerod, 1994).

In contrast, in cells exposed to 50 μM palmitic acid, there were progressively more cells in $G_0/G_1$ phase and fewer cells in S-phase, while the proportion of cells in the $G_2$/M-phase remained static (Figure 6), suggesting that the cells are entering the plateau phase of growth. This is apparently inconsistent with Figure 1 where cells exposed to 50 μM palmitic acid appear to be still in the logarithmic phase of growth. The reasons for such inconsistencies have been discussed in detail (Baserga, 1995) and include the observation that changes in cell cycle precede any effects on cell number. The cell doubling time is also dependent on the entire cell cycle profile and the time that cells spend in each phase. In addition, the calculation of percentage of cells within each phase of the cell cycle is based on a best-fit model to estimate the proportion of cells in each phase and this may introduce further inaccuracies when comparisons are made between cell cycle data and cell number data.

The growth of a tumour depends on the balance between proliferation by mitosis and cell loss through necrosis or apoptosis. From the results of this study, EPA seems to exert an effect on both proliferation and apoptosis. EPA appears to inhibit proliferation by arresting cells in the cell cycle and increases cell loss by the induction of apoptosis. In the present study apoptosis accounts for significant cell loss. The induction of apoptosis may explain the increase in tumour cell loss observed in transplantable tumour models following oral administration of menhaden oil and EPA (Hudson *et al.*,

1993; Gabor and Abraham, 1986). Although we cannot conclude from the present study that cell cycle arrest and the induction of apoptosis are exclusive mechanisms involved in the observed effects of EPA on various tumour cell lines *in vitro* (Beck *et al*., 1991; Lystad *et al*., 1994) and tumour growth *in vivo* (Hudson *et al*., 1993; Tisdale and Beck, 1991), the two mechanisms would appear to make a significant contribution. We have also performed DNA analysis on two other pancreatic cancer cell lines exposed to EPA and obtained similar findings with the appearance of hypodiploid peaks (data not shown) which suggest that EPA-induced apoptosis is not a unique feature of MIA PaCa-2 cells.

Further studies are required if EPA is to be used as an anti-tumour agent in a clinical context. The mechanism by which EPA induces cell cycle arrest and apoptosis and methods of enhancing its effect on the induction of apoptosis require investigation. It has been suggested that EPA, as a competitive inhibitor of arachidonic acid, may reduce the production of eicosanoids such as prostaglandin $E_2$ which are essential for the survival of tumour cells (Karmali *et al*., 1984, 1989). This hypothesis is not supported by the observation that cyclooxygenase inhibitors like indomethacin are unable to influence the growth of tumour cells *in vivo* (Feldman and Hilf, 1985; Abou-El-Ela *et al*., 1989). In addition, the effects of indomethacin on various *in vitro* models show that it can either stimulate or inhibit tumour cell proliferation (Buckman *et al*., 1991; Fulton, 1984; Rose and Connolly, 1990; Bayer *et al*., 1979; Hial *et al*., 1977). Other studies have suggested that increased lipid peroxidation may be an important cause of cytotoxicity associated with *n*-3 PUFAs but a definite role has not been established (Falconer *et al*., 1994; Lystad *et al*., 1994).

Various reports in the literature have shown a link between cell surface receptors and their associated kinases and the induction of apoptosis (Kleinman *et al*., 1994; Spinozzi *et al*., 1994). Most of the incorporated EPA appears to remain in the phospholipid component of cell membranes (Brown and Subbaiah, 1994), although the cytoplasmic lipid accumulation observed in the present study may suggest that other mechanisms are also important. However, it has been shown that the relative quantity of polyunsaturated fatty acids in the cell membrane may play an important role in regulation of proliferation and cellular functions (Brown and Subbaiah, 1994; Pan *et al*., 1990; Bandyophadhyay *et al*., 1993). It is possible that EPA may alter the microenvironment of surface receptors or signalling proteins in the cell membrane, thereby inducing inappropriate signalling moieties and initiating cell cycle arrest and apoptosis.

Further experimental work on the pharmacokinetics of EPA, mode of delivery to the tumour and mechanism of EPA-induced apoptosis will provide information which will be useful in designing regimens for the treatment of pancreatic cancer.

**Acknowledgements**
This work was supported by the University of Edinburgh Cancer Research Fund and in part by the Edinburgh Royal Infirmary Cancer Research Fund and by CRC grant SP2142/0101.

### References

ABOU-EL-ELA SH, PRASSE KW, FARREL RL, CARROL RW, WADE AE AND BUNCE OR. (1989). Effect of D,L-2-difluoroethylornithine and indomethacin on mammary tumour promotion in rats fed high n-3 and/or n-6 fat diets. *Cancer Res*., **49**, 1434–1440.

ARENDS MJ AND WYLIE AH. (1991). Apoptosis: mechanisms and role in pathology. *Int. Rev. Exp. Pathol*., **32**, 223–256.

BAGWELL CB, HUDSON JL AND IRVIN GL III. (1979). Nonparametric flow cytometry analysis. *J. Histochem. Cytochem*., **27**, 293–296.

BANDYOPHADHYAY GK, HWANG S, IMAGAWA W AND NANDI S. (1993). Role of polyunsaturated fatty acids as signal transducers: amplification of signals from growth factor receptors by fatty acids in mammary epithelial cells. *Prostaglandins Leukot. Essent. Fatty Acids*, **48**, 71–78.

BASERGA R. (1995). Measuring parameters of growth. In *Cell Growth and Apoptosis – A Practical Approach*, 1st ed. Studzinski GP (ed.) pp 12–17. Oxford University Press: Washington.

BAYER BM, KRUTH HS, VAUGHAN M AND BEAVEN MA. (1979). Arrest of cultured cell in the G1 phase of the cell cycle by indomethacin. *J. Pharmacol. Exp. Ther*., **210**, 106–111.

BECK SA, SMITH KL AND TISDALE MJ. (1991). Anti-cachetic and anti-tumour effect of eicosapentaenoic acid and its effect on protein turnover. *Cancer Res*., **51**, 6089–6093.

BEGIN ME, ELLS G, DAS UN AND HORROBIN DF. (1986). Differential killing of human carcinoma cells supplemented with n-3 and n-6 polyunsaturated fatty acids. *J. Natl Cancer Inst*., **77**, 1053–1062.

BROWN ER AND SUBBAIAH PV. (1994). Differential effects of eicosapentaenoic acid and docosahexaenoic acid on human skin fibroblasts. *Lipids*, **29**, 825–829.

BRYSON GJ, HARMON BV AND COLLIN RJ. (1994). A flow cytometric study of cell death: failure of some models to correlate with morphological assessment. *Immunol. Cell Biol*., **72**, 35–41.

BUCKMAN DK, HUBBARD NE AND ERIKSON KL. (1991). Eicosanoids and linoleated-enhanced growth of mouse mammary tumour cells. *Prostaglandins Leukot. Essent. Fatty Acids*, **44**, 177–184.

CALDER PC, BOND JA, SAMANTHA JB, HUNT SV AND NEWSHOLME EA. (1991). Effect of fatty acids on the proliferation of concanavalin A-stimulated rat lymph node lymphocytes. *Int. J. Biochem*., **23**, 579–588.

CARTER DC. (1989). Cancer of the pancreas. *Curr. Opin. Gastroenterol*., **5**, 716–722.

COHEN JJ. (1991). Programmed cell death in the immune system (review). *Adv. Immunol*., **50**, 55–85.

COHEN JJ AND DUKE RC. (1984). Glucocorticoid activation of a calcium dependent endonuclease in thymocyte nuclei leads to cell death. *J. Immunol*., **132**, 38–42.

DEMARCO DM, SANTOLI D AND ZURIER RB. (1994). Effects of fatty acids on proliferation and activation of human synovial compartment lymphoctes. *J. Leukocyte Biol*., **56**, 612–615.

FALCONER JS, ROSS JA, FEARON KCH, HAWKINS RA AND CARTER DC. (1994). Effect of eicosapentaenoic acid and other free fatty acids on the growth *in vitro* of human pancreatic cancer cell lines. *Br. J. Cancer*, **69**, 826–832.

FELDMAN JM AND HILF R. (1985). Failure of indomethacin to inhibit growth of the R323OAC mammary tumour in rats. *J. Natl Cancer Inst*., **75**, 751–756.

FULTON AM. (1984). *In vivo* effects of indomethacin on the growth of murine mammary tumours. *Cancer Res*., **44**, 2416–2420.

GABOR H AND ABRAHAM S. (1986). Effect of dietary menhaden oil on tumour cell loss and the accumulation of mass of a transplantable mammary adenocarcinoma in BALB/c mice. *J. Natl Cancer Inst*., **76**, 1223–1229.

GREENSPAN P, MAYER EP AND FOWLER SD. (1985). Nile red: a selective fluorescent stain for intracellular lipid droplets. *J. Cell Biol*., **100**, 965–973.

HIAL V, DEMELLO MCF, HORAKOVA Z AND BEAVEN MA. (1977). Anti-proliferative activity of anti-inflammatory drugs in two mammalian cell culture lines. *J. Pharmacol. Exp. Ther*., **202**, 446–454.

HOLLEY RW, BALDWIN JH AND KIRENAN JA. (1974). Control of growth of a tumour by linoleic acid. *Proc. Natl Acad. Sci. USA*, **71**, 3976–3978.

HUDSON EA, BECK SA AND TISDALE MJ. (1993). Kinetics of the inhibition of tumour growth in mice by eicosapentaenoic acid – reversal by linoleic acid. *Biochem. Pharmacol*., **45**, 2189–2194.

KARMALI RA, MARSH J AND FUCHS, C. (1984). Effects of omega-3 fatty acids on growth of a rat mammary tumour. *J. Natl Cancer Inst*., **73**, 457–461.

KARMALI RA, CHAO C, BASU A AND MODAK M. II. (1989). Effects of n-3 fatty acids on mammary H-ras expression and $PGE_2$ levels in DMBA-treated rats. *Anticancer Res.*, **9,** 1169 – 1174.

KLEINMAN D, DOUVDEVANI A, SCHALLY AV, LEVY J AND SHARONI Y. (1994). Direct growth inhibition of human endometrial cancer by the gonadotropin-releasing hormone antagonist SB-75: role of apoptosis. *Am. J. Obstet. Gynecol.*, **170,** 96 – 102.

LYSTAD E, HOSTMARK AT, KISERUD C, PATRICK KE AND FISHER SM. (1994). Influence of fatty acids and bovine serum albumin on the growth of human hepatoma and immortalised human kidney epithelial cells. *In vitro Cell Dev. Biol.*, **30A,** 568 – 573.

MIDDLETON PG, MILLER S, ROSS JA, STEEL CM AND GUY K. (1992). Insertion of SMRV-H viral DNA at the c-*myc* gene locus of a Burkitt's lymphoma cell line and presence in established cell lines. *Int. J. Cancer*, **52,** 451 – 454.

MINOURA T, TAKATA T, SAKAGUCHI M, TAKADA H, YAMAMURA M, HIOKI K AND YAMAMOTO M. (1988). Effect of dietary eicosapentaenoic acid on azoxymethane-induced colon carcinogenesis in rats. *Cancer Res.*, **48,** 4790 – 4794.

ORMEROD MG. (1994). In *Flow Cytometry – a Practical Approach* Omerod MG (ed.), pp. 130 – 135. Oxford University Press: Washington.

PALMER KR, KERR M KNOWLES G, CULL A, CARTER DC and LEONARD RCF. (1994). Chemotherapy prolongs survival in inoperable pancreatic carcinoma. *Br. J. Surg.*, **81,** 882 – 885.

PAN DA, SULLIVAN-TAILYOUR G AND HULBERT AJ. (1990). Membrane fatty acid changes during the cell cycle of CV-1 cells. *Exp. Cell Res.*, **191,** 141 – 143.

ROSE DP AND CONNOLLY JM. (1990). Effects of fatty acids and inhibitors on eicosanoids synthesis on the growth of a human breast cancer cell line in culture. *Cancer Res.*, **50,** 7139 – 7144.

SOYLAND E, LEA T, SANDSTAD B AND DREVON A. (1994). Dietary supplementation with very long chain n-3 fatty acids in man decreases expression of the interleukin-2 receptor (CD25) on mitogen-stimulated lymphocytes from patients with inflammatory skin diseases. *Eur. J. Clin. Invest.*, **24,** 236 – 242.

SPINOZZI F, PAGLIACCI MC, MIGLIORATI G, MORACA R, GRIGNANI F, RICCARDI C AND NICOLETTI I. (1994). The natural tyrosine kinase inhibitor genisten produces cell cycle arrest and apoptosis in Jurkat T-leukemia cells. *Leuk. Res.*, **18,** 431 – 439.

TATE GA, MANDELL BF, KARMALI RA, LAPOSATA M, BAKER DG, SCHUMACHER HR AND ZURIER RB. (1988). Suppression of monosodium urate crystal-induced acute inflammation by diets enriched with gammalinolenic acid and eiscosapentaenoic acid. *Arthritis Rheum.*, **31,** 1543 – 1551.

TELFORD WG, KING LE AND FRAKER PJ. (1994). Rapid quantification of apoptosis in pure and heterogenous cell populations using flow cytometry. *J. Immunol. Methods*, **172,** 1 – 16.

TISDALE MJ AND DHESI JK. (1990). Inhibition of weight loss by omega-3 fatty in an experimental cachexia model. *Cancer Res.*, **50,** 5022 – 5026.

TISDALE MJ AND BECK SA. (1991). Inhibition of tumour-induced lipolysis *in vitro* and cachexia and tumour growth *in vivo* by eicosapentaenoic acid. *Biochem. Pharmacol.*, **41,** 103 – 107.

WYLIE AH, KERR JFR AND CURRIE AR. (1990). Cell death: the significance of apoptosis. *Int. Rev. Cytol.*, **68,** 251 – 306.


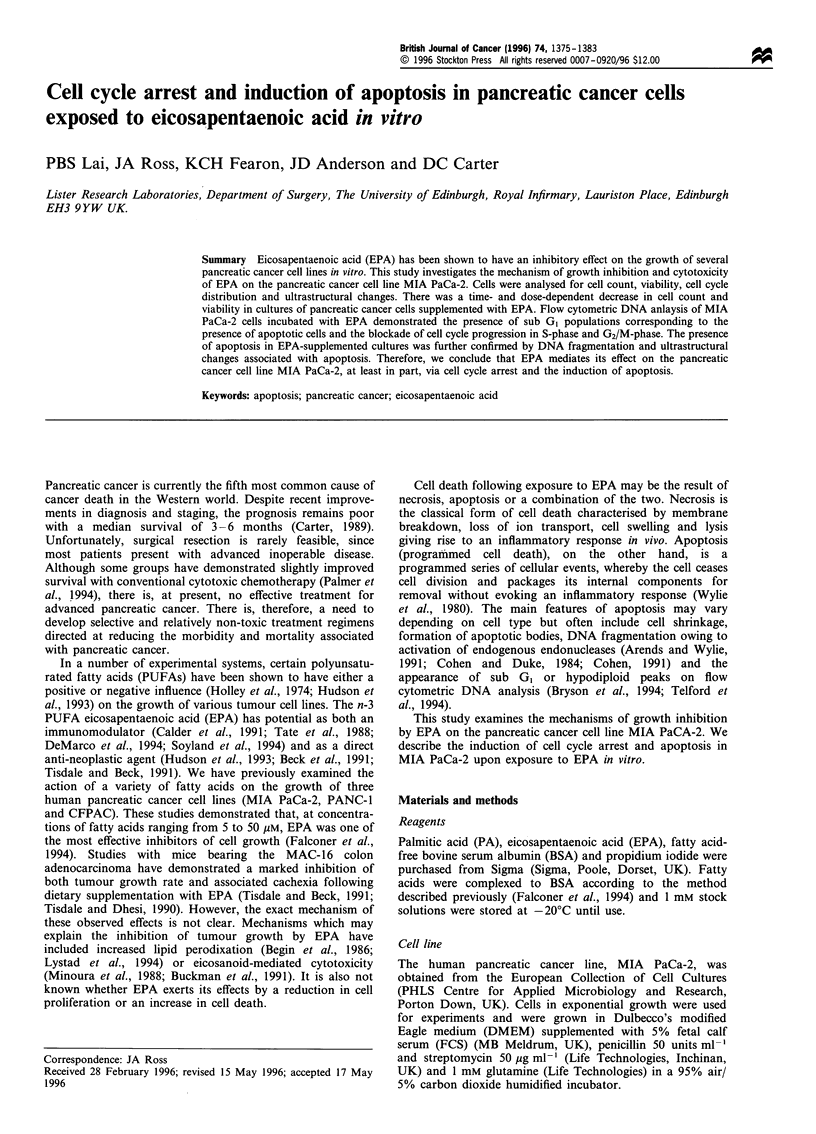

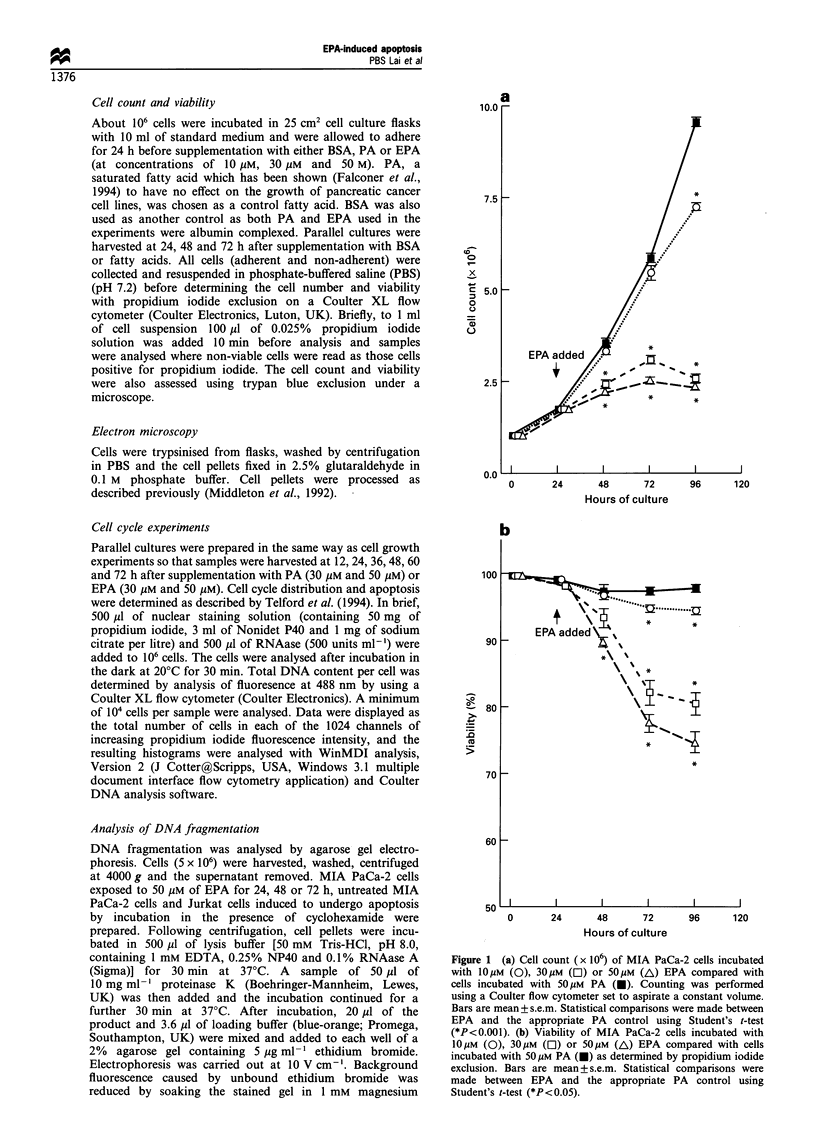

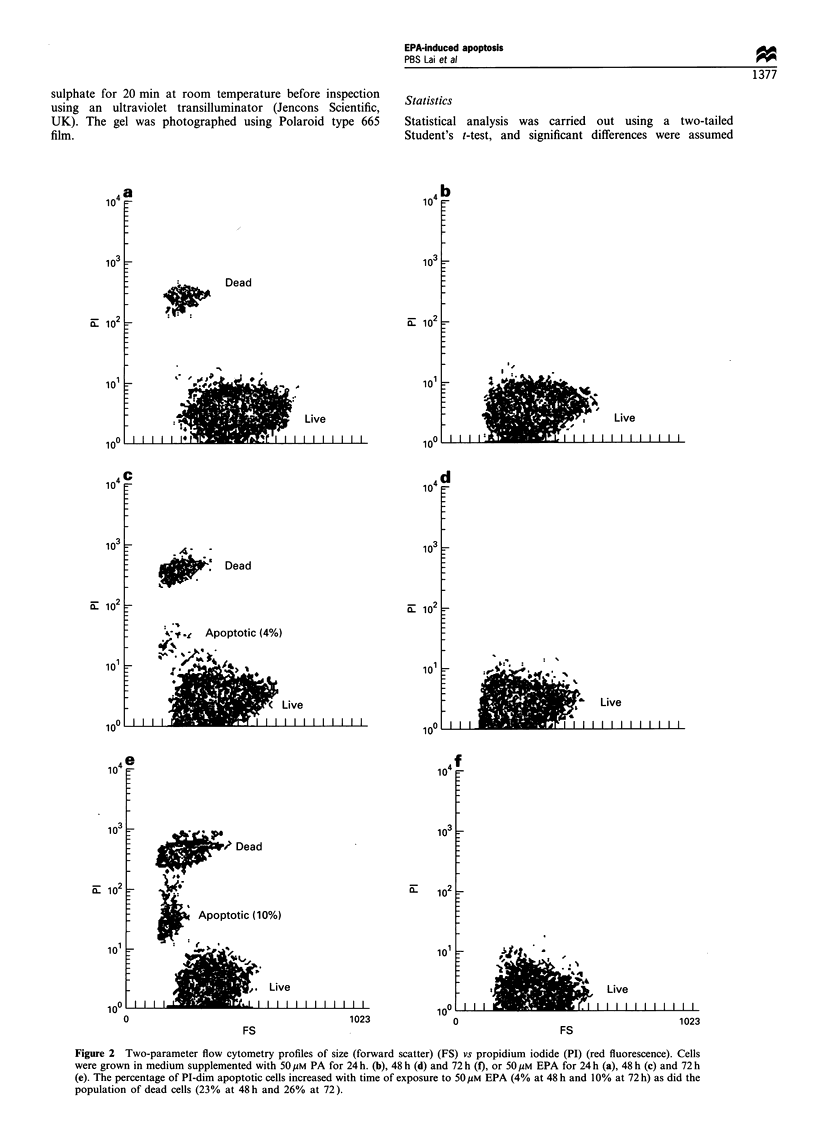

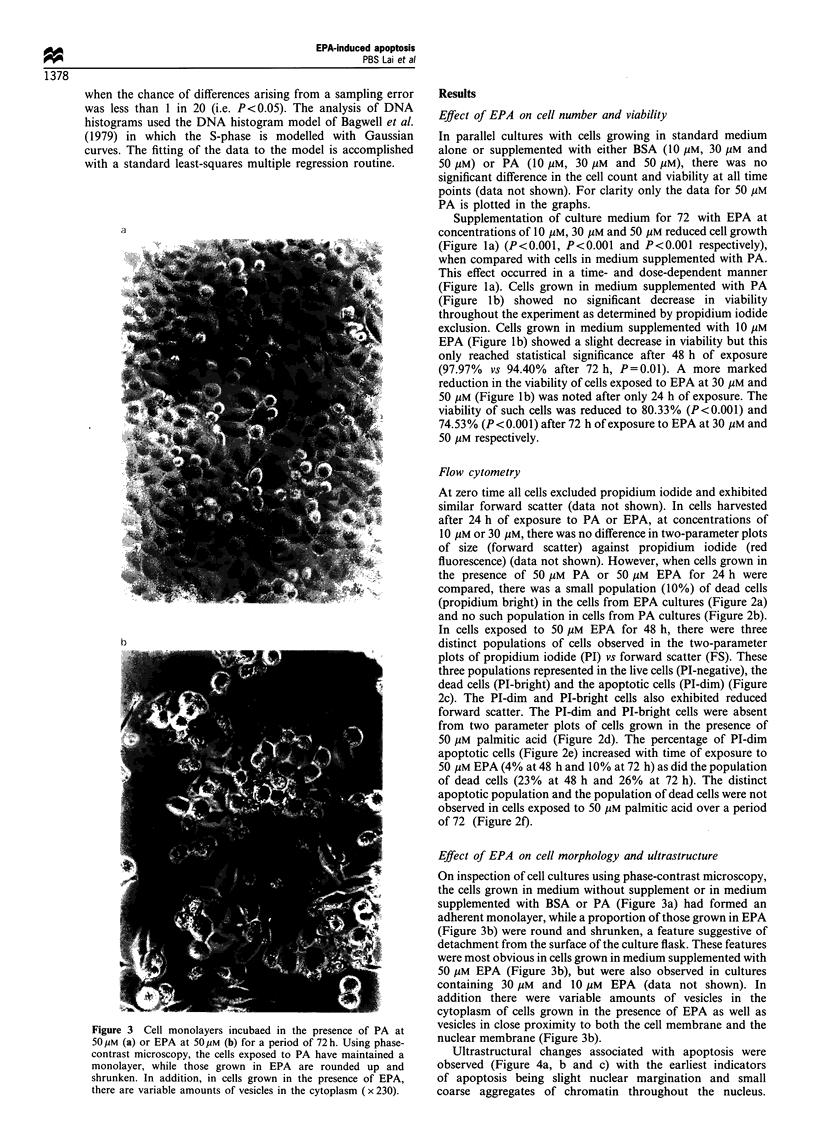

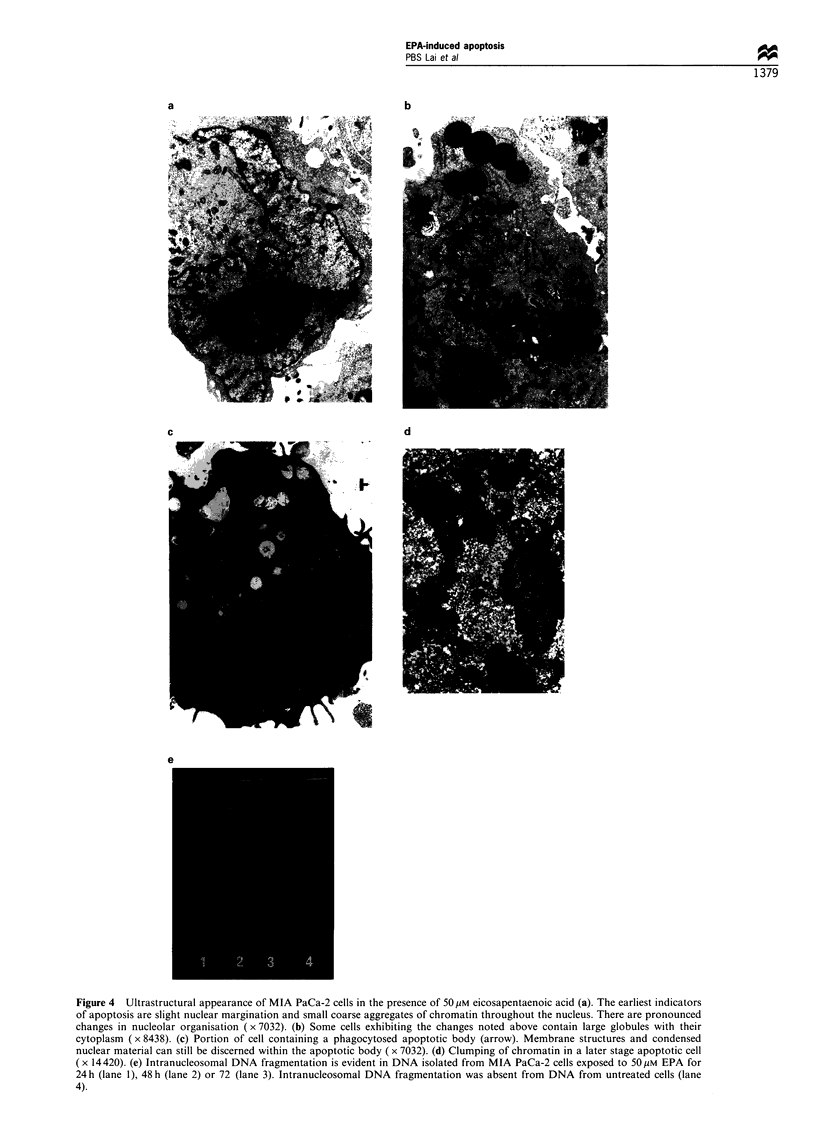

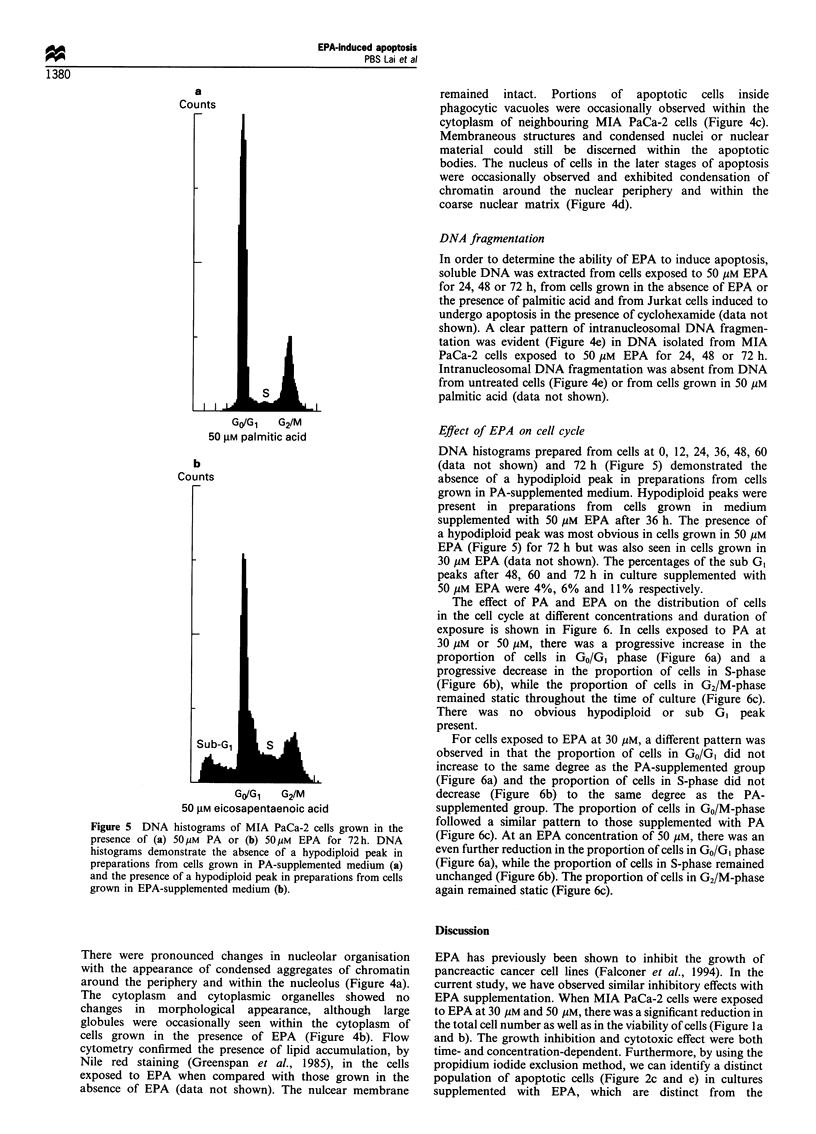

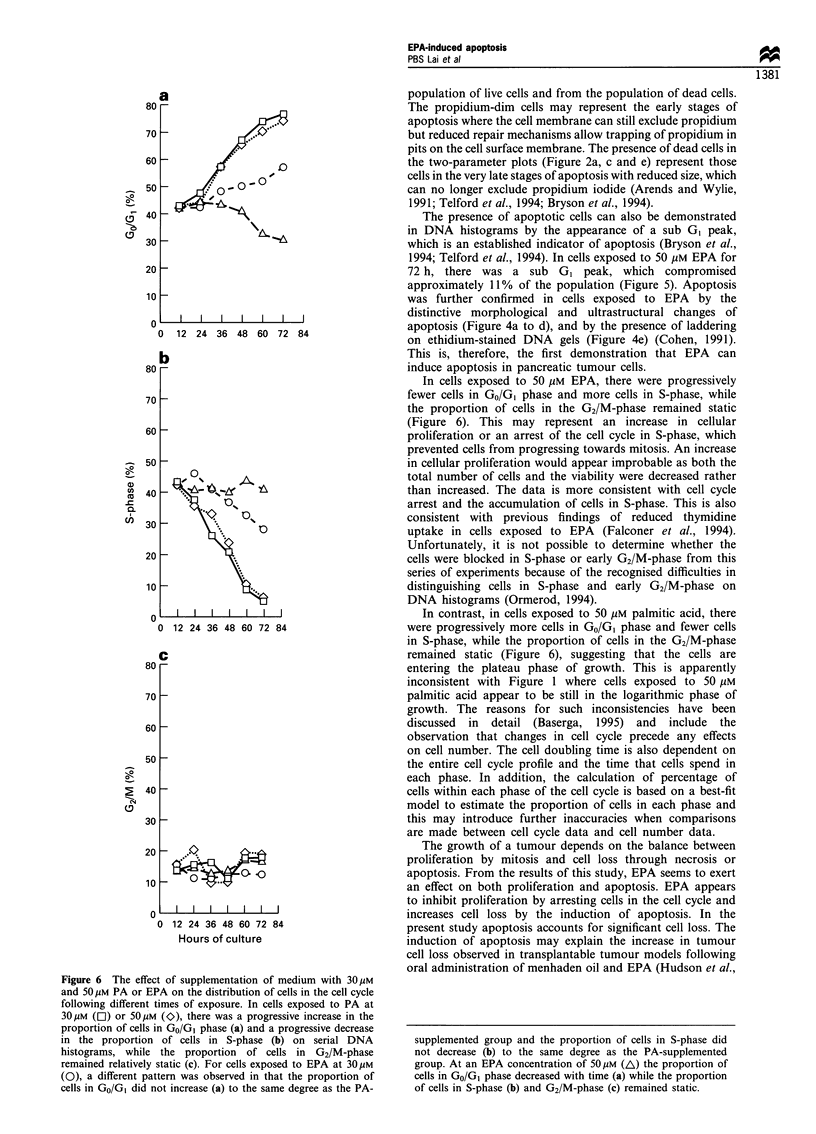

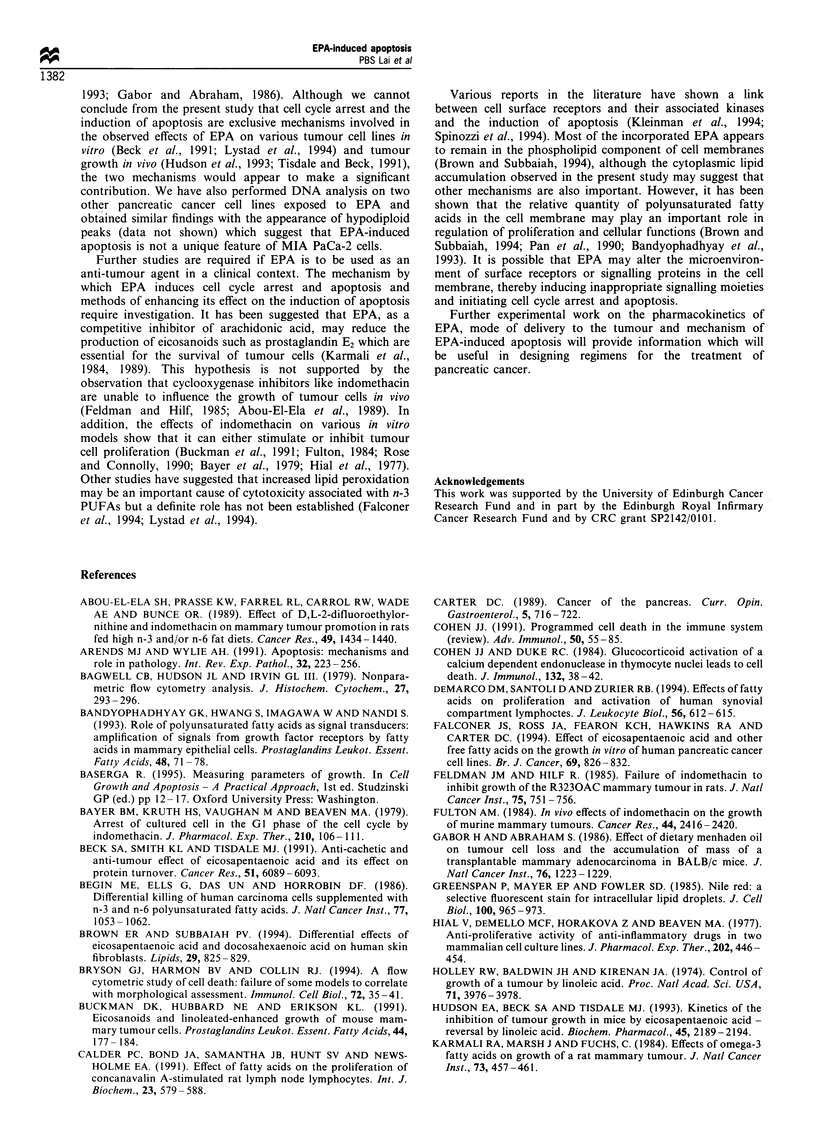

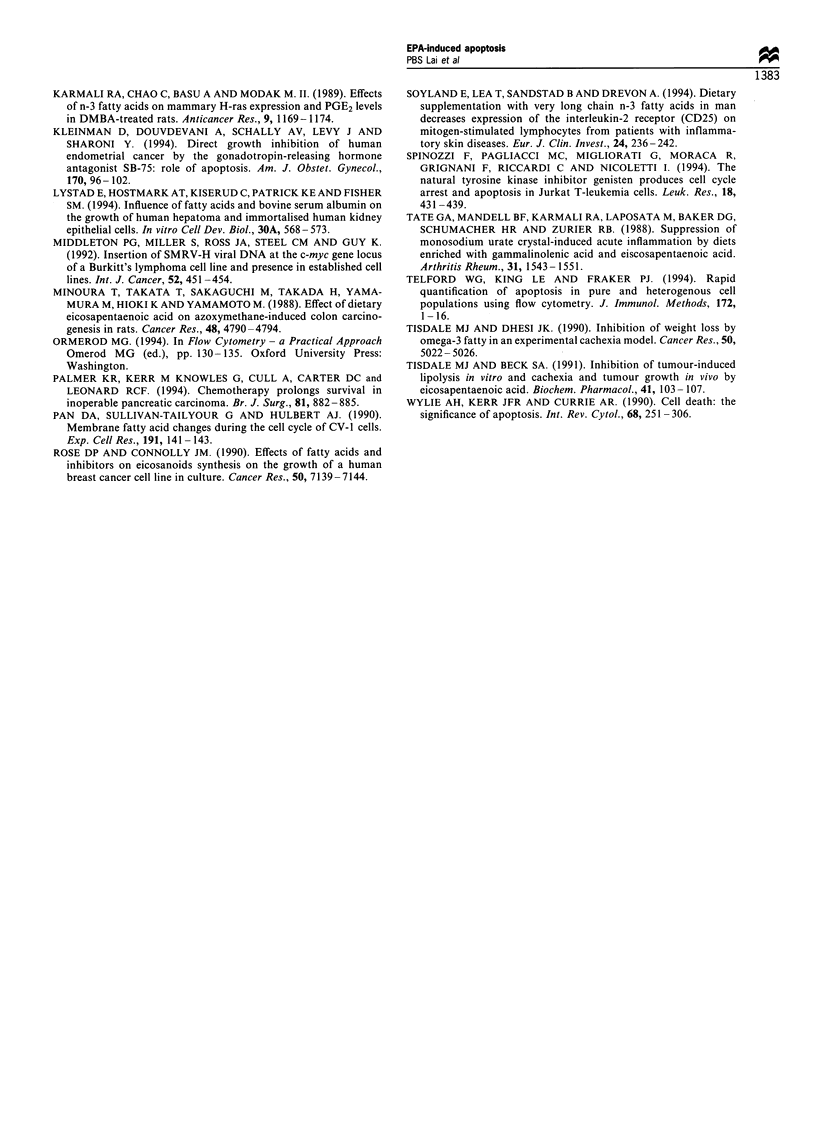


## References

[OCR_00990] Abou-el-Ela S. H., Prasse K. W., Farrell R. L., Carroll R. W., Wade A. E., Bunce O. R. (1989). Effects of D,L-2-difluoromethylornithine and indomethacin on mammary tumor promotion in rats fed high n-3 and/or n-6 fat diets.. Cancer Res.

[OCR_00993] Arends M. J., Wyllie A. H. (1991). Apoptosis: mechanisms and roles in pathology.. Int Rev Exp Pathol.

[OCR_00999] Bagwell C. B., Hudson J. L., Irvin G. L. (1979). Nonparametric flow cytometry analysis.. J Histochem Cytochem.

[OCR_01004] Bandyopadhyay G. K., Hwang S., Imagawa W., Nandi S. (1993). Role of polyunsaturated fatty acids as signal transducers: amplification of signals from growth factor receptors by fatty acids in mammary epithelial cells.. Prostaglandins Leukot Essent Fatty Acids.

[OCR_01014] Bayer B. M., Kruth H. S., Vaughan M., Beaven M. A. (1979). Arrest of cultured cells in the G1 phase of the cell cycle by indomethacin.. J Pharmacol Exp Ther.

[OCR_01019] Beck S. A., Smith K. L., Tisdale M. J. (1991). Anticachectic and antitumor effect of eicosapentaenoic acid and its effect on protein turnover.. Cancer Res.

[OCR_01030] Brown E. R., Subbaiah P. V. (1994). Differential effects of eicosapentaenoic acid and docosahexaenoic acid on human skin fibroblasts.. Lipids.

[OCR_01037] Bryson G. J., Harmon B. V., Collins R. J. (1994). A flow cytometric study of cell death: failure of some models to correlate with morphological assessment.. Immunol Cell Biol.

[OCR_01040] Buckman D. K., Hubbard N. E., Erickson K. L. (1991). Eicosanoids and linoleate-enhanced growth of mouse mammary tumor cells.. Prostaglandins Leukot Essent Fatty Acids.

[OCR_01024] Bégin M. E., Ells G., Das U. N., Horrobin D. F. (1986). Differential killing of human carcinoma cells supplemented with n-3 and n-6 polyunsaturated fatty acids.. J Natl Cancer Inst.

[OCR_01046] Calder P. C., Bond J. A., Bevan S. J., Hunt S. V., Newsholme E. A. (1991). Effect of fatty acids on the proliferation of concanavalin A-stimulated rat lymph node lymphocytes.. Int J Biochem.

[OCR_01060] Cohen J. J., Duke R. C. (1984). Glucocorticoid activation of a calcium-dependent endonuclease in thymocyte nuclei leads to cell death.. J Immunol.

[OCR_01056] Cohen J. J. (1991). Programmed cell death in the immune system.. Adv Immunol.

[OCR_01065] DeMarco D. M., Santoli D., Zurier R. B. (1994). Effects of fatty acids on proliferation and activation of human synovial compartment lymphocytes.. J Leukoc Biol.

[OCR_01073] Falconer J. S., Ross J. A., Fearon K. C., Hawkins R. A., O'Riordain M. G., Carter D. C. (1994). Effect of eicosapentaenoic acid and other fatty acids on the growth in vitro of human pancreatic cancer cell lines.. Br J Cancer.

[OCR_01078] Feldman J. M., Hilf R. (1985). Failure of indomethacin to inhibit growth of the R3230AC mammary tumor in rats.. J Natl Cancer Inst.

[OCR_01083] Fulton A. M. (1984). In vivo effects of indomethacin on the growth of murine mammary tumors.. Cancer Res.

[OCR_01087] Gabor H., Abraham S. (1986). Effect of dietary menhaden oil on tumor cell loss and the accumulation of mass of a transplantable mammary adenocarcinoma in BALB/c mice.. J Natl Cancer Inst.

[OCR_01091] Greenspan P., Mayer E. P., Fowler S. D. (1985). Nile red: a selective fluorescent stain for intracellular lipid droplets.. J Cell Biol.

[OCR_01096] Hial V., De Mello M. C., Horakova Z., Beaven M. A. (1977). Antiproliferative activity of anti-inflammatory drugs in two mammalian cell culture lines.. J Pharmacol Exp Ther.

[OCR_01104] Holley R. W., Baldwin J. H., Kiernan J. A. (1974). Control of growth of a tumor cell by linoleic acid.. Proc Natl Acad Sci U S A.

[OCR_01109] Hudson E. A., Beck S. A., Tisdale M. J. (1993). Kinetics of the inhibition of tumour growth in mice by eicosapentaenoic acid-reversal by linoleic acid.. Biochem Pharmacol.

[OCR_01124] Karmali R. A., Chao C. C., Basu A., Modak M. (1989). II. Effect of n-3 and n-6 fatty acids on mammary H-ras expression and PGE2 levels in DMBA-treated rats.. Anticancer Res.

[OCR_01114] Karmali R. A., Marsh J., Fuchs C. (1984). Effect of omega-3 fatty acids on growth of a rat mammary tumor.. J Natl Cancer Inst.

[OCR_01130] Kleinman D., Douvdevani A., Schally A. V., Levy J., Sharoni Y. (1994). Direct growth inhibition of human endometrial cancer cells by the gonadotropin-releasing hormone antagonist SB-75: role of apoptosis.. Am J Obstet Gynecol.

[OCR_01136] Lystad E., Høstmark A. T., Kiserud C., Haugen A. (1994). Influence of fatty acids and bovine serum albumin on the growth of human hepatoma and immortalized human kidney epithelial cells.. In Vitro Cell Dev Biol Anim.

[OCR_01142] Middleton P. G., Miller S., Ross J. A., Steel C. M., Guy K. (1992). Insertion of SMRV-H viral DNA at the c-myc gene locus of a BL cell line and presence in established cell lines.. Int J Cancer.

[OCR_01146] Minoura T., Takata T., Sakaguchi M., Takada H., Yamamura M., Hioki K., Yamamoto M. (1988). Effect of dietary eicosapentaenoic acid on azoxymethane-induced colon carcinogenesis in rats.. Cancer Res.

[OCR_01160] Palmer K. R., Kerr M., Knowles G., Cull A., Carter D. C., Leonard R. C. (1994). Chemotherapy prolongs survival in inoperable pancreatic carcinoma.. Br J Surg.

[OCR_01164] Pan D. A., Sullivan-Tailyour G., Hulbert A. J. (1990). Membrane fatty acid changes during the cell cycle of CV-1 cells.. Exp Cell Res.

[OCR_01169] Rose D. P., Connolly J. M. (1990). Effects of fatty acids and inhibitors of eicosanoid synthesis on the growth of a human breast cancer cell line in culture.. Cancer Res.

[OCR_01179] Spinozzi F., Pagliacci M. C., Migliorati G., Moraca R., Grignani F., Riccardi C., Nicoletti I. (1994). The natural tyrosine kinase inhibitor genistein produces cell cycle arrest and apoptosis in Jurkat T-leukemia cells.. Leuk Res.

[OCR_01172] Søyland E., Lea T., Sandstad B., Drevon A. (1994). Dietary supplementation with very long-chain n-3 fatty acids in man decreases expression of the interleukin-2 receptor (CD25) on mitogen-stimulated lymphocytes from patients with inflammatory skin diseases.. Eur J Clin Invest.

[OCR_01188] Tate G. A., Mandell B. F., Karmali R. A., Laposata M., Baker D. G., Schumacher H. R., Zurier R. B. (1988). Suppression of monosodium urate crystal-induced acute inflammation by diets enriched with gamma-linolenic acid and eicosapentaenoic acid.. Arthritis Rheum.

[OCR_01193] Telford W. G., King L. E., Fraker P. J. (1994). Rapid quantitation of apoptosis in pure and heterogeneous cell populations using flow cytometry.. J Immunol Methods.

[OCR_01204] Tisdale M. J., Beck S. A. (1991). Inhibition of tumour-induced lipolysis in vitro and cachexia and tumour growth in vivo by eicosapentaenoic acid.. Biochem Pharmacol.

[OCR_01199] Tisdale M. J., Dhesi J. K. (1990). Inhibition of weight loss by omega-3 fatty acids in an experimental cachexia model.. Cancer Res.

[OCR_01209] Wyllie A. H., Kerr J. F., Currie A. R. (1980). Cell death: the significance of apoptosis.. Int Rev Cytol.

